# Supplementary figures and images for: Curcumin-Loaded Self-Assembly Constructed by Octenylsuccinate Fish (Cyprinus carpio L.) Scale Gelatin: Preparation and Characterization
Source: Foods. 2022 Sep 19;11(18):2911. doi: 10.3390/foods11182911 (PMC9498313; doi:10.3390/foods11182911)

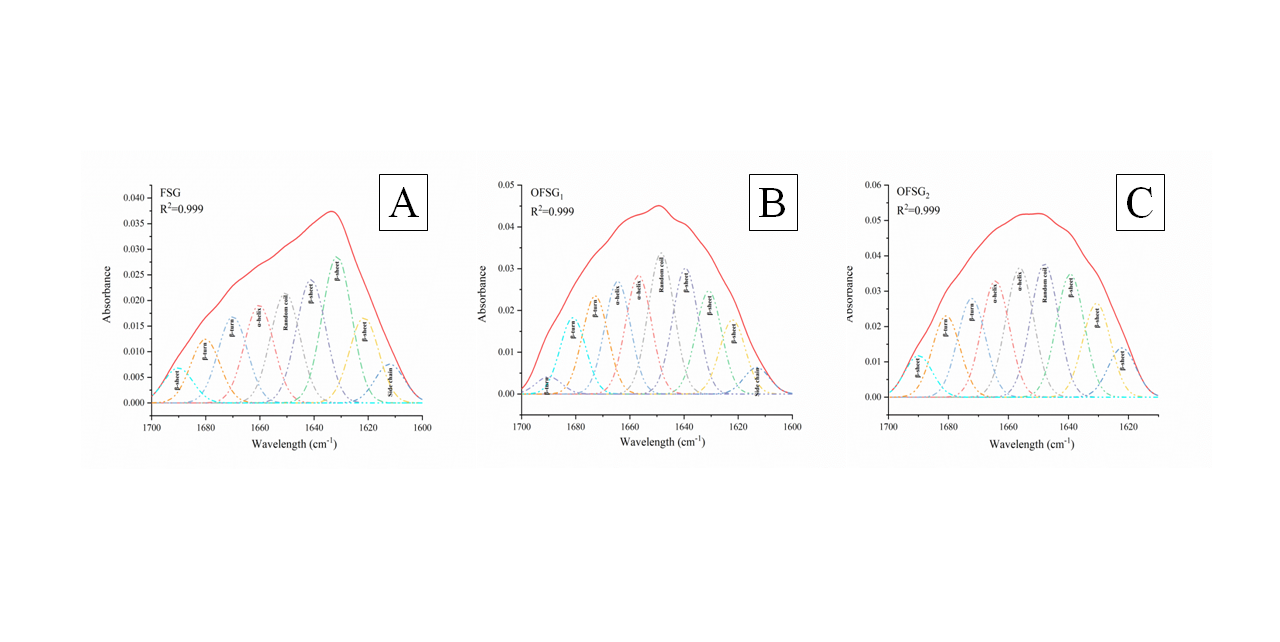

Supplement: Supplementary file 1 [file foods-11-02911-s001.zip › foods-1851608-supplementary.tif]
